# Towards realistic benchmarks for multiple alignments of non-coding sequences

**DOI:** 10.1186/1471-2105-11-54

**Published:** 2010-01-26

**Authors:** Jaebum Kim, Saurabh Sinha

**Affiliations:** 1 Department of Computer Science, University of Illinois at Urbana-Champaign, Urbana, IL 61801, USA; 2 Institute for Genomic Biology, University of Illinois at Urbana-Champaign, Urbana, IL 61801, USA

## Abstract

**Background:**

With the continued development of new computational tools for multiple sequence alignment, it is necessary today to develop benchmarks that aid the selection of the most effective tools. Simulation-based benchmarks have been proposed to meet this necessity, especially for non-coding sequences. However, it is not clear if such benchmarks truly represent real sequence data from any given group of species, in terms of the difficulty of alignment tasks.

**Results:**

We find that the conventional simulation approach, which relies on empirically estimated values for various parameters such as substitution rate or insertion/deletion rates, is unable to generate synthetic sequences reflecting the broad genomic variation in conservation levels. We tackle this problem with a new method for simulating non-coding sequence evolution, by relying on genome-wide distributions of evolutionary parameters rather than their averages. We then generate synthetic data sets to mimic orthologous sequences from the *Drosophila *group of species, and show that these data sets truly represent the variability observed in genomic data in terms of the difficulty of the alignment task. This allows us to make significant progress towards estimating the alignment accuracy of current tools in an absolute sense, going beyond only a relative assessment of different tools. We evaluate six widely used multiple alignment tools in the context of *Drosophila *non-coding sequences, and find the accuracy to be significantly different from previously reported values. Interestingly, the performance of most tools degrades more rapidly when there are more insertions than deletions in the data set, suggesting an asymmetric handling of insertions and deletions, even though none of the evaluated tools explicitly distinguishes these two types of events. We also examine the accuracy of two existing tools for annotating insertions versus deletions, and find their performance to be close to optimal in *Drosophila *non-coding sequences if provided with the true alignments.

**Conclusion:**

We have developed a method to generate benchmarks for multiple alignments of *Drosophila *non-coding sequences, and shown it to be more realistic than traditional benchmarks. Apart from helping to select the most effective tools, these benchmarks will help practitioners of comparative genomics deal with the effects of alignment errors, by providing accurate estimates of the extent of these errors.

## Background

The availability of genome sequences of closely related species (such as 18 placental mammal species [1] and 12 *Drosophila *species [2]) has provided opportunities to solve several key biological problems such as the inference of phylogenetic trees, reconstruction of ancestral genomes, estimation of evolutionary rates, identification of conserved and non-conserved regions, and more generally the study of genome structure and evolution. The alignment of multiple sequences, highlighting regions of homology among the sequences and predicting nucleotide level relationships among them, plays a critical role in such analyses. Numerous attempts have been made to develop accurate and efficient methods to solve the multiple sequence alignment problem (reviewed in [3-6]), offering us much flexibility, as well as difficulty, in choosing the most appropriate tool(s) for the task. Another important task related to multiple alignment is the annotation of insertions and deletions (indels) in the alignment, a task that has received some attention in recent years [7-12] in light of the realization that indels may be responsible for genomic variation as much as nucleotide substitutions are [13], and that indels may affect regional mutation rates [14].

Given the availability of multiple tools to perform either of these two tasks, a researcher faces two important questions: "Which of the tools should I use for my task?" and "How accurate will the tool be on my data?" Answers to these come from studies that use data sets ("benchmarks") where the true answers are known, to evaluate and compare different tools. The design of benchmarks therefore directly affects the reliability of bioinformatics analyses that use those tools. The two most widely used benchmarking approaches for alignment tools are (i) to make use of biological sequences and their manually curated alignments from databases such as Homstrad [15], BAliBASE [16], and SABmark [17], or (ii) to simulate the evolution of biological sequences by using specialized tools such as Dawg [18], Rose [19] and INDELible [20]. The main advantage of the former approach is the use of real biological sequences and alignments that are produced by using protein structure information. This approach does not apply to non-coding DNA sequences, whose alignments form the basis of regulatory comparative genomics. Therefore, simulation-based benchmarks have been widely adopted in this context [21-26]. The simulation approach, however, is highly dependent on its parameters that reflect the underlying evolutionary processes and their rates. It is not clear how to choose "correct" settings for these parameters and how to assess if the simulated sequences mimic real data well enough for claims about alignment accuracy, both in relative terms (i.e., comparison of tools) and in the absolute, to generalize from the benchmarks to the real world setting. We address these questions in this work, whose main contributions are the following.

1) We present a new simulation-based benchmarking method that is based on the entire spectrum of values of its parameters as inferred from real data. This is in contrast to existing approaches that rely on the average observed values of the parameters.

2) We quantify the difficulty of aligning a data set by leveraging recent developments [27] on estimating alignment accuracy without requiring the "true" alignments. We reason that if the synthetic data sets truly mimic real orthologous sequences, the difficulty of aligning them ought to match that for the real data. This is the key insight used to determine how realistic a particular benchmark (i.e., collection of data sets) is, and we use this idea to show that the novel simulation method produces far more realistic benchmarks than the existing approach.

3) Using our new benchmarks, we evaluate and compare the accuracy of six multiple alignment tools (ClustalW [28], Dialign-TX [29], Mafft [30], Mavid [31], Mlagan [32], and Pecan [33]) on *Drosophila *non-coding sequences. The specific alignment task we consider is that of global alignment of ~1-10 Kbp long sequences, and our conclusions may not apply to the task of local alignment, which was studied in [21]. We are able to estimate the accuracy of alignment for specific sets of *Drosophila *genomes, and find these to be very different from previously reported values. We also evaluate two schemes for annotating insertions and deletions specifically, and find their accuracy to be comparable, and close to optimal.

4) We find that data sets with an excess of deletions over insertions are more amenable to accurate alignment than those with an excess of insertions, suggesting an implicit bias (in the alignment tools) with respect to their treatment of indels, even though none of the evaluated tools explicitly makes a distinction between insertions and deletions.

## Results

### Simulation of non-coding sequences by a traditional method

Modeling of DNA sequence evolution has been studied extensively in the past, and state-of-the-art simulation programs [18-20] draw on various aspects of such models. Simulation of non-coding sequences [21] incorporates current understanding of the architecture of such sequences in terms of regions of evolutionary constraint, for example by stipulating the presence of short (but variable length) subsequences that evolve at a much slower rate than the rest of the sequence. We refer the reader to [18,21] for a comprehensive description of these approaches, which form the foundation of our own work reported here. These simulation programs rely crucially on the values of their parameters (e.g., substitution rate or frequency of constrained blocks). The parameters serve to fully specify the stochastic processes from which evolutionary events (e.g., substitutions or indels) will be sampled, and prescribe the *expected *frequency of those events in the generated data sets. Variation in the frequency of these events, which underlie the difficulty of alignment tasks, results from the inherent randomness of the simulation process, i.e., the differences in random choices made from one "run" of the process to another. It is natural to ask if the resulting variability across data sets in a synthetic benchmark is comparable to the corresponding variability observed in real orthologous sequences. The question is particularly relevant due to the heterogeneity of non-coding sequences with respect to the density of functional elements and also motivated by the known variation in evolutionary rates across loci [34-36].

We began by implementing the above-mentioned simulation paradigm, which we call the "traditional" paradigm, by incorporating the "constraint blocks" idea of Pollard et al. [21] into the Dawg simulation program [18]. Parameters, including phylogeny, branch lengths, indel frequency, and various parameters related to conserved blocks were set based on previously published values from the literature [21,37] or estimated by us from published multiple alignments of *Drosophila *non-coding sequences (see Methods). A key difference in our implementation was that branch lengths (i.e., average substitution rates) were estimated from non-coding sequences themselves, instead of synonymous substitution rates from coding sequences, as has been done previously. We elaborate on this important issue later in this section.

**Figure 1:**
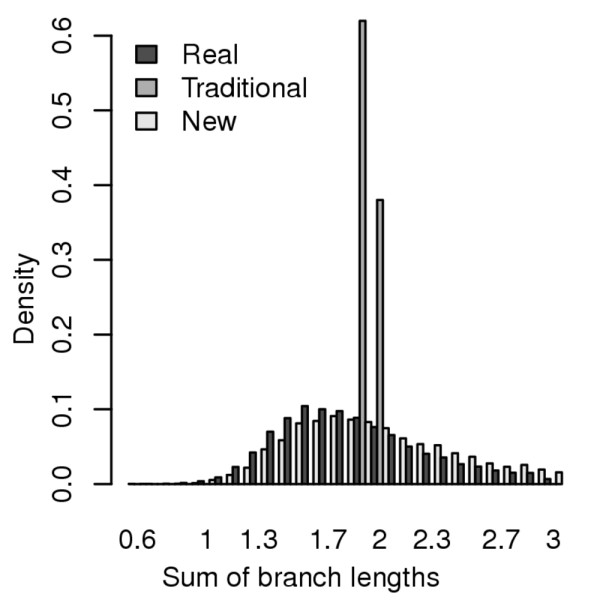
**Distributions of sum of branch lengths in a phylogenetic tree estimated from real data and synthetic data respectively**. Sequences of eight *Drosophila *species were collected from real data ("Real"), data produced by a traditional simulator ("Traditional"), and data produced by the new simulator based on parameter sampling ("New"). The traditional simulator used the average substitution rates observed in the real data, while the new simulator used the empirical distribution of substitution rates in real data. The branch lengths were estimated by Paml [51].

We considered the alignments of real *Drosophila *sequences from eight species (see Methods), computed the sum of branch lengths of the phylogenetic tree estimated from ~1 Kbp segments of alignment, and found the distribution of this statistic to have a large variance across the genome (black bars in Figure 1). The same distribution, when computed from 100 synthetic data sets generated using the traditional simulator described above, and the same alignment program, shows a very sharp peak around the mean (dark gray bars in Figure 1). We note that the means of the two distributions are similar (1.87 in real data and 1.94 in synthetic data), since the benchmark was parameterized by the average substitution rates observed in real data. This is the first clear evidence that existing simulators fall short of representing the *range *of conservation levels in real data.

**Figure 2:**
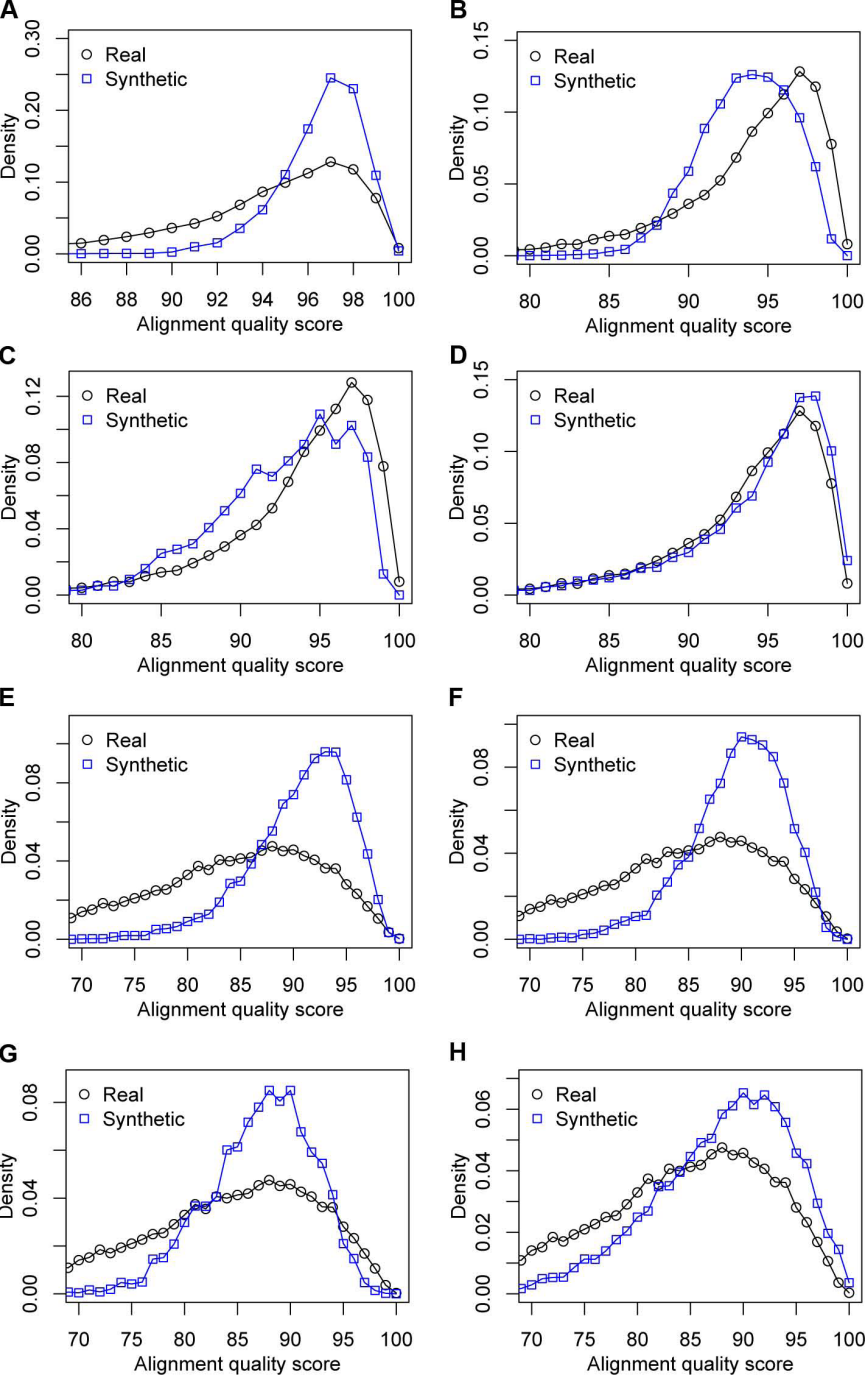
**Distributions of alignment quality scores - HoT SPS (A-D) and HoT CS (E-H) - between real and simulated sequences**. Synthetic sequences were simulated by (A, E) a traditional method, (B, F) using a mixture model of evolutionary rates, (C, G) using a mixture model of ratios of substitutions to indels, and (D, H) a novel method that relies on observed genome-wide distributions of its parameters.

Since substitution rates are generally correlated with indel rates, a large variance in the former implies a corresponding variance in indel frequencies, which of course lie at the root of the alignment problem. This suggests that if we could measure the "difficulty of alignment" in any region of the genome (e.g., by having knowledge of the true alignment, and measuring the accuracy of a powerful alignment program), we ought to see a large variability in this measure across the genome. Moreover, if the observed distribution of the alignment difficulty measure is comparable to that in a benchmark, we would be confident in making claims about performance of alignment tools based on that benchmark. The problem is that measuring alignment difficulty on real data requires knowledge of their true alignment, which is unavailable. Recent work by Landan and Graur [27] showed that a reasonable surrogate for the accuracy of an alignment program on a data set can be computed even without the true alignment. They reasoned that good alignments should be invariant to the *orientation *of the input sequences, and therefore defined the "Heads or Tails (HoT)" alignment quality score as the agreement between two alignments, one generated from original sequences and the other from their reversed versions. Hall [38] later showed that there is a clear positive correlation between HoT alignment quality scores and the real alignment accuracy measured by comparison with the true alignment. This remarkable finding inspired us to formulate the following strategy for quantifying the spectrum of alignment difficulty in data sets. We computed the HoT alignment quality score on the computed alignment of a data set, and used this score as a surrogate for the alignment difficulty of the data set. (The alignment was computed using a well-established alignment program called Pecan [33], but other choices would not affect our conclusions.) Low values of the alignment quality score indicate that the data set is particularly hard to align, and high values are suggestive of an "easy" data set. As shown in Figure 2A, the distributions of the score were significantly different between synthetic and real data sets. Alignment quality scores for 83% of the synthetic sequences are above 95, whereas close to 50% of real sequences had scores below this range. This strongly suggests that by and large the synthetic sequences simulated by the traditional approach are easier to align than real sequences, even though the former were generated with evolutionary parameters mirroring their real data counterparts. In particular, the variance of alignment quality (and presumably of alignment difficulty) is much smaller in synthetic data sets.

### Simulation based on a mixture model of parameters

We hypothesized that the above observation about synthetic data sets was due to the use of a single setting of the branch lengths, and the relatively low variability resulting from the randomness of the process itself (Figure 1). If this is true, then one way to alleviate the problem would be to allow for multiple phylogenies for simulation of different data sets, with the variability of branch lengths across phylogenies introducing an additional source of data set variability. We therefore considered a set of *K = 10 *phylogenies {*ϕ*_*1*_, *ϕ*_*2*_, ..., *ϕ*_*K*_} that are scaled versions of the original phylogeny *ϕ*_*0*_, i.e., every branch length in phylogeny *ϕ*_*i *_is a constant factor *τ*_*i *_times the corresponding branch length in *ϕ*_*0*_. (We used {*τ*_*i*_} = {1,2, ..., 10}.) We modified the simulator to first sample at random one of the *K *phylogenies, and simulate according to this setting of branch lengths, with all other parameters being fixed as before. In other words, the distribution of alignment quality scores from the new simulation process is a mixture distribution, with components parameterized by different phylogenies and the probability of sampling any particular phylogeny being the mixture weight. We estimated an upper bound on the agreement between this mixture distribution and the observed distribution of alignment quality scores, by maximum likelihood training of mixture weights, through expectation-maximization algorithm [39]. This "best fit" mixture distribution is shown in Figure 2B, along with the real data distribution, and reveals a much stronger agreement between the two distributions, as compared to Figure 2A. The same trend was seen when allowing for a set of values of the "substitution to indel ratio" parameter (with values 10:1,10:2, ..., 10:5), keeping all other parameters, including the phylogeny, fixed (Figure 2C). These results strongly suggested that the use of a range of parameter values instead of a single value has great impact on the variability of alignment difficulty in synthetic data sets, and has the potential to lead to the generation of realistic sequences.

### Simulation based on parameter sampling

The above results, while encouraging in terms of better reproducing the genomic variability of alignment difficulty, were obtained by fitting parameters of the simulation process so as to best match real data. We next asked if we could achieve the same or better agreement between the synthetic and real data distributions without having seen the real distribution of alignment quality scores. This would then allow us to use the observed agreement as a relatively unbiased assessment of how realistic the benchmark is. Developing the mixture model idea from the previous section, we now computed for each parameter the entire distribution of values observed in real data alignments, just as the traditional approach estimates the average of these values. The simulation process was now made to sample each parameter independently from its empirical distribution, and then generate a data set based on the sampled parameter values. The benchmark thus constructed (comprising 10000 different data sets) was examined for its distribution of alignment quality scores, and as seen in Figure 2D, this distribution was remarkably close to that observed in real sequences. In other words, the newly constructed benchmark meets our pre-specified criterion for a "realistic" benchmark. (It also shows strong agreement, as expected, with real data in terms of estimated branch lengths; Figure 1.)

The above analysis was performed using the sum-of-pairs score (SPS), which is the simplest of the scores defined in the HoT approach [27]. We repeated all analyses with another score, called the HoT column score (CS), and observed the same trends (Figure 2E-H), although the agreement between synthetic and real data distributions was not as strong now as with the SPS (Figure 2D) (also see Discussion).

### Assessment of multiple alignment tools

#### Accuracy of multiple alignments

**Figure 3:**
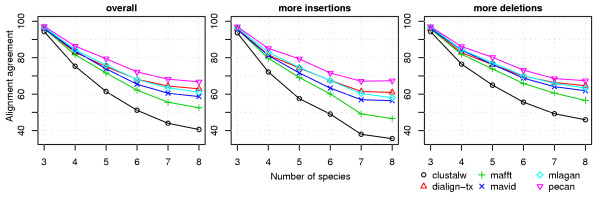
**Performance of multiple alignment tools compared by alignment agreement**. The scores were calculated by using all synthetic data sets (left panel), and by using only data sets where the expected number of insertions is two times more than the number of deletions or vice versa (middle and right panels respectively).

We used our new benchmark to evaluate and compare six leading multiple alignment tools that are publicly available and can align DNA sequences. These are ClustalW 2.0.5 [28], Dialign-TX 1.0.0 [29], Mafft 6.240 [30], Mavid 2.0 build 4 [31], Mlagan 2.0 [32], and Pecan 0.7 [33]. We performed the assessment with varying numbers of species, *K *= 3, ..., 8. For each choice of *K*, 10000 sets of sequences corresponding to *K *different *Drosophila *species were simulated and the above alignment tools were run with default parameters or with the best setting recommended by their authors. We then compared the resulting alignments to the "true" alignments reported by the simulation program, using the following three commonly used evaluation measures [40,41]: (i) *alignment agreement*, which is the fraction of aligned base pairs (or bases aligned to gaps) in the predicted alignment that agree with the true alignment, (ii) *alignment sensitivity*, which is the fraction of aligned base pairs of the true alignment that agree with the predicted alignment, and (iii) *alignment specificity*, which is the fraction of aligned base pairs of the predicted alignment that agree with the true alignment. Whereas the alignment agreement score considers aligned base pairs as well as bases aligned to gaps, the sensitivity and specificity scores are calculated *only *from aligned base pairs. The results of our evaluations are shown in Figure 3 and Additional files 1 and 2 (left panels) (see Additional file 3 for an example of true and computed alignments by the six alignment programs). The Pecan alignment program was found to be superior by all three measures, across all values of *K*. Its performance degrades more slowly (with increasing *K*) than the other tools, as a result of which the gap between Pecan and the other tools became larger more species were included in the tests. The average alignment agreement in five species alignments produced by Pecan (the species most divergent from *D. melanogaster *being *D. pseudoobscura*) was close to 80%, but degraded to ~67% when aligning all eight species.

We performed the same evaluations by limiting ourselves to those data sets (in the benchmark) that had an excess of insertions over deletions, and separately to those data sets with an excess of deletions (Figure 3, and Additional files 1 and 2; middle and right panels). Surprisingly, we saw a clear difference between these two classes of data sets, with most tools performing significantly worse when there was an excess of insertions in the data set. For example, on data sets with *K = 8*, ClustalW showed an alignment agreement of 36% or 46% depending on whether there was an excess of insertions or deletions (respectively). The same trend was seen in terms of the alignment sensitivity and specificity measures. Noticably, Pecan was largely unaffected by this dichotomy of data sets. (For additional insights on how alignment accuracy depends on various other descriptive statistics of a data set, e.g., total divergence, indel count, or total indel length, see Additional file 4.)

**Figure 4:**
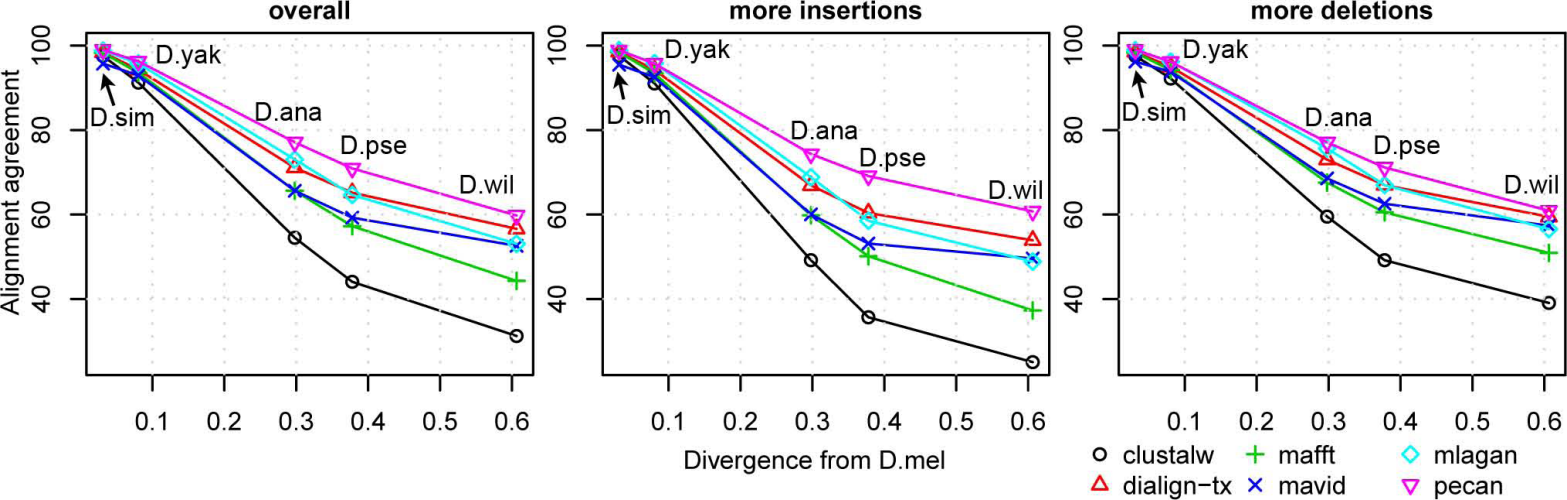
**Performance of multiple alignment tools compared by alignment agreement of pairs of species**. The scores were calculated by using all synthetic data sets (left panel), and by using only data sets where the expected number of insertions is two times more than the number of deletions or vice versa (middle and right panels respectively).

The evaluation measures used above consider all pairs of species in the *K*-species alignment and sum the accuracy values obtained from all pairs, without regard to the varying divergences of different pairs. In an attempt to address this issue, we separately measured the alignment accuracy of different pairs of species (e.g., *D. melanogaster *- *D. simulans*, *D. melanogaster - D. yakuba*, etc.), limiting ourselves to the eight-species data sets. All trends reported above were also seen in this alternative view of the results (Figure 4, and Additional files 5 and 6). The alignment agreement, using Pecan, for *D. melanogaster *with *D. yakuba*, *D. anannassae*, *D. pseudoobscura *and *D. willistoni *was found to be 96%, 77%, 71% and 60% respectively.

#### Disagreement with estimates based on existing benchmark

**Figure 5:**
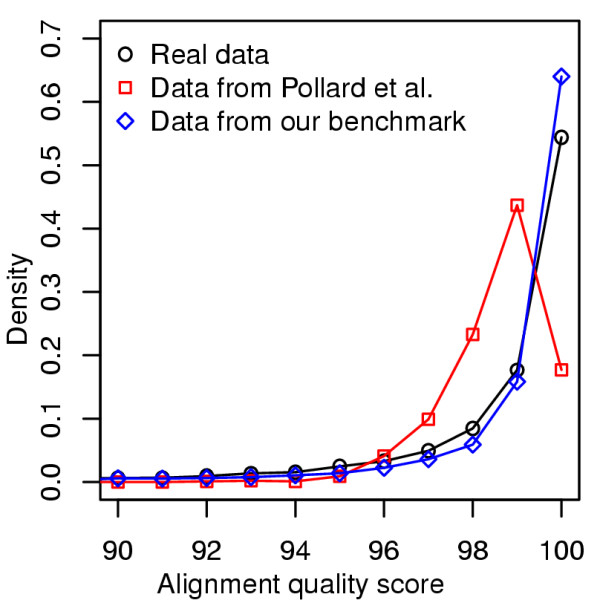
**Distributions of alignment quality scores of data sets representing *D. melanogaster - D. pseudoobscura *pair from real genomes, Pollard et al. **[21]**, and our benchmark**. The collected data sets from each of the three sources were aligned by Pecan [33] and then their alignment quality scores were calculated by HoT SPS [27] method.

We found a substantial disagreement between our performance estimates and those previously reported by Pollard et al. [21] using their own benchmark. For instance, the alignment sensitivity for the *D. melanogaster *- *D. pseudoobscura *pair comes out to be ~70% in our assessment and ~40% by their estimates, using the Mlagan alignment tool. We observe such gaps (with higher numbers in our benchmark) also for alignment specificity, and for other species pairs and alignment programs as well (Additional files 7 and 8). (We confirmed this by evaluating the alignment programs ourselves on the Pollard et al. [21] benchmark, see Methods.) While this discordance could be in part due to the fact that our benchmark employs a spectrum of parameter values to achieve greater and more realistic variability, we believe the major difference here is that even the average substitution rate, a key parameter in both simulation programs, is widely different between their study and ours. The estimate used by Pollard et al. [21] (~2.4 substitutions per site) is based on silent positions in codons, while our estimate (~0.38 substitutions per site) reflects the average subsitution frequency (between these species) seen in non-coding sequences. In light of the results of Figure 2D, where we show that our benchmark accurately mirrors the range of alignment difficulty in real data, the use of non-coding sequences in estimating this key parameter seems better justified. We investigated this issue with additional tests. We collected data sets representing the *D. melanogaster - D. pseudoobscura *pair from Pollard et al. [21], as well as from our benchmark and the real genomes. The alignment quality score (HoT SPS) distributions were computed for each type of benchmark, and are shown in Figure 5. We observed a close agreement between our data sets and the real orthologous sequences, while the Pollard et al. [21] data sets were harder to align on average, consistent with the greater substitution rate used there. As noted in Methods, the overall substitution frequency observed in non-coding sequences may be viewed as an average of the corresponding frequency in conserved blocks and the much higher frequency outside conserved blocks. This average is determined by two key parameters *α*, the fraction of sequence length that falls into conserved blocks, and *β*, the ratio of the evolutionary rate of conserved blocks to that outside blocks. Given that the divergence estimate used by Pollard et al. [21] for these two species is ~2.24 (median) substitutions per site, if we are to treat this value as the neutral rate (i.e., rate outside conserved blocks) in non-coding sequences, what values of *α *and *β *would lead to the observed overall substitution frequency of 0.38? We determined that if *β *= 0.1, as was used by Pollard et al. [21] (and also by us), *α *has to be ~0.92, i.e., about 92% of non-coding sequences have to be conserved blocks, which is far higher than most current estimates of this parameter [37,42]. Similarly, if we are to trust the values of *α *= 0.2 and *β *= 0.1, as was used by Pollard et al. [21] (and also by us, based on estimates from real data), then the overall divergence, after averaging between conserved blocks and non-blocks, would be ~1.84 substitutions per site, far greater than what is observed (0.38). We therefore concluded that the use of synonymous substitution rates as the neutral rate for non-coding sequence is likely to lead to benchmarks with overly "diverged" sequences that are more difficult to align than real sequences from those species.

### Assessment of indel annotation schemes

Traditional alignment programs mark the predicted locations of insertions and deletions as "gaps", and do not proceed to annotate these gaps as being insertions or deletions. This latter task has received some attention recently with at least two "indel annotation schemes" being published, based on maximum-parsimony ("sbInfer" [7]) and probabilistic-models ("Indelign" [12]) respectively. We examined the accuracy of these two alignment-related tools on our new benchmark. (Indelign was modified for additional efficiency, see Methods.) We noted that the best alignment agreement score (among all methods, as shown in Figure 4) is ~70% for *D. melanogaster - D. pseudoobscura*, and decreases to ~60% when a more diverged species (*D. willistoni*) is added. Reasoning that phylogenies for which computed alignments are largely inaccurate would not be suitable for insertion/deletion annotation in any case, we chose to limit our assessment to the following five *Drosophila *species: *D. melanogaster*, *D. simulans*, *D. yakuba*, *D. ananassae*, and *D. pseudoobscura *(see Additional file 9 for phylogeny). The "true" alignment (as indicated by the simulation program) was provided to the two indel annotation tools and the insertion/deletion annotations on each of the five terminal branches (leading to the extant species) of the phylogeny were compared to the "true" annotations. The following three measures were used for assessment, borrowed from [12]: (i) *Indel Count Agreement*, which is the agreement of indel counts between true and predicted annotations, (ii) *Indel Ratio Agreement*, which is the agreement of the ratio of the number of insertions to the total number of indels between the two annotations, and (iii) *Indel Annotation Coverage*, which is the fraction of indel positions on which the two annotations agree (see Methods). (Both sensitivity and specificity scores were calculated for the Indel Annotation Coverage.)

As summarized in Table 1, Indel Count Agreement scores of the two tools were very similar to each other and close to optimal (0) for most species except *D. pseudoobscura*, the species with the longest terminal branch in the phylogeny. Indel Ratio Agreement scores of both tools were close to optimal (1) in all five species. While the sensitivity scores of Indel Annotation Coverage of the two tools were above 90% across all five species, the specificity scores were above 90% only for the four species except *D. pseudoobscura*. The loss of accuracy on the *D. pseudoobscura *branch is presumably due to the fact that there is no "outgroup" species to aid disambiguation of insertions and deletions on this branch. We further discuss the implications of these observations in the next section. We also repeated our assessment for sequences with an excess of insertions or of deletions, as above, but no significant differences was observed between these two categories (data not shown).

**Table 1:** Performance of indel annotation tools compared by different measures (ICA, IRA, IAC) on five-species alignments.

	ICA^a^		IRA^b^		IAC^c ^(sensitivity)		IAC^c ^(specificity)	
**Species**	**Indelign**	**sbInfer**	**Indelign**	**sbInfer**	**Indelign**	**sbInfer**	**Indelign**	**sbInfer**

D. sim	0.06	0.06	1.00	1.01	0.97	0.96	0.99	0.99

D. mel	0.04	0.04	1.00	1.01	0.99	0.99	0.99	0.98

D. yak	0.06	0.05	1.00	1.01	0.98	0.97	0.97	0.98

D. ana	0.08	0.07	1.00	1.00	0.93	0.91	0.93	0.96

D. pse	0.24	0.27	1.02	1.03	0.94	0.96	0.79	0.79

^a^Indel Count Agreement (optimal value = 0)

^b^Indel Ratio Agreement (optimal value = 1)

^c^Indel Annotation Coverage (optimal value = 1)

## Discussion

Choosing the most suitable tool for aligning orthologous sequences is essential to studies in comparative genomics and in molecular evolution, making it critical to develop accurate benchmarking methodology. In this study, we propose a novel simulation-based approach to generate realistic data sets mimicking orthologous non-coding sequences from multiple *Drosophila *species. This new simulation method exploits the spectrum of values of evolutionary statistics (e.g., substitution rate, indel frequency) seen across a genome. We take advantage of an objective "alignment quality" measure to show that the synthetic sequences produced agree with real sequences not only in terms of evolutionary statistics, but are also as easy or hard to align as real data sets. In this sense, our evaluation results are more likely to reflect the actual accuracy values of alignment-related tools on data from *Drosophila *species. We note that our strategy of sampling parameters (used in evolutionary simulations) from their empirical distribution has parallels with traditional Bayesian inference where one integrates over (i.e., samples from) a prior distribution on parameters, rather than using a single point estimate.

A key step in our benchmark construction was the ability to assess the quality of an alignment without access to the corresponding true alignment. This ability has been the result of several recent publications by other authors. Prakash and Tompa [43,44] developed statistical methods to assess if a multiple sequence alignment appears contaminated with one or more unrelated sequences, based on which they identified regions of whole genome alignments as being suspect. The development of the "HoT" method by Landan and Graur [27] then came as a breakthrough to assess the reliability of multiple sequence alignments. Later on, Landan and Graur [45] extended the HoT method to take advantage of co-optimal alternative alignments generated by progressive alignment tools. However, the implementation of this method is too dependent on the specific procedures of a progressive alignment method, making the original HoT score [27] a natural choice for our purpose.

While our benchmark is shown to be very close to real sequences in terms of the distribution of HoT SPS, we are cautioned by the discrepancy observed between simulated and real sequences in terms of the HoT CS, an alternative alignment quality score from the same authors (Figure 2E-H). This is likely the product of properties of non-coding sequences that are not adequately represented in our simulation process. For example, modeling the functional constraints embedded in non-coding sequences through short conserved blocks (with scaled down phylogenies) is surely an oversimplification of the complexity of genomic architecture. Important progress has been made on this front, in the form of specialized evolutionary simulators that model transcription factor binding site evolution in realistic ways [24,46,47]. Each of these simulators makes specific assumptions about *cis*-regulatory architecture, vis-a-vis the density and evolution of binding sites. However, it is not yet clear which, if any, of these different assumed models of regulatory sequence evolution is most suited to represent the variability in constraint patterns across different regions of the genome. Our simplistic "conserved block" model (borrowed from [37]) seems to be a good approximation that captures the most prominent patterns in orthologous non-coding sequences, in terms of alignment difficulty. We expect that future research on more realistic models of *cis*-regulatory architecture will lead us to replace the alternating arrangement of conserved blocks and faster evolving segments with a pattern more in line with reality. Future work may also include careful modeling of genomic repeats and repeat generating evolutionary events, since repeat-rich genomes may present additional challenges for the alignment task. Our proposed framework of sampling evolutionary parameters before running the simulation process will remain equally important in future benchmarks that implement such sophisticated models.

Some clarification is in order with respect to our manner of choosing substitution rates for the simulation process, since it marks a significant departure from traditional thinking. The latter, as embodied in the work of Pollard et al. [21], prescribes that the "unconstrained" parts of the sequence evolve with nucleotide substitution rate equal to that infered from synonymous mutations in the nearby gene (or average over all genes). This rate (~2.4 substitutions/site for *D. melanogaster *- *D. pseudoobscura*) is widely different from the value observed in real non-coding sequence alignments (~0.4 substitutions/site). One could argue that this gap may be offset if we set an appropriate frequency of conserved positions (with very low rates), resulting in an average substitution rate that is close to the empirically observed value. However, this turned out not be the case for any realistic setting of the frequency of conserved positions (data not shown). We therefore chose to be guided by existing estimates of the frequency and length distribution of conserved blocks, with substitution rates that are some constant *β *(see Methods) times the "neutral" rate outside of the blocks, and set this neutral rate so that the average rate for the entire sequence matches observed values. Our choice reflects the philosophy that simulated data sets ought to match real data in terms of various evolutionary statistics and net alignment difficulty, and the discordance of the used neutral rate from synonymous substitution rates is ignored for the sake of practicality.

To our knowledge, no previous benchmarking study has evaluated the effect of insertions and deletions on the performance of alignment tools. Some studies [21-25] have used equal frequencies for insertions and deletions and focused on the collective effects of indels. Here, we attempted to elucidate the differing effects of insertions and deletions by separately summarizing results for the two extreme cases where the number of insertions is at least two times the frequency of deletions and vice versa. The results were surprising, and indicated that most multiple alignment tools find it harder to accurately align data sets with an excess of insertions than those with more deletions (Figures 3 and 4). Löytynoja and Goldman [48] offered valuable insight into a possible source of this asymmetry, pointing out that progressive alignment methods (a category to which all the methods tested here belong) "end up penalizing single insertion events multiple times". We speculate therefore, as they did, that claims about insertion/deletion frequencies along the genome should be preceded by an examination of the alignment method's accuracy in regimes of high insertion frequency.

Finally, a note about our findings on insertion/deletion annotation. Indelign [12] is a probabilistic tool that annotates insertions and deletions by maximum likelihood training of an evolutionary model. sbInfer [7] is a greedy algorithm that reconstructs ancestral sequences based on the maximum parsimony principle, and therefore allows us to infer insertion/deletion annotations. To assess these two tools without being confounded by errors of an alignment program, we examined their performance on the true alignments. We found the two programs to have comparable accuracy on our benchmark for the five *Drosophila *species. While the accuracy was close to optimal on four of the five terminal branches, we observed that both tools over-estimate insertions as well as deletions on the longest branch (leading *to D. pseudoobscura*), while accurately predicting the ratio of insertions to deletions. We note that the *D. pseudoobscura *branch in the phylogenetic tree originates from the root of the tree, and we would expect to have better annotation results for this branch if an appropriate outgroup species was used. For studies that intend to use insertion to deletion ratio profiling to identify loci with unusual evolutionary patterns (e.g., [9]) it may be safe to examine all five terminal branches of this tree; however, for the more common requirement of accurately annotating insertion and deletion events, e.g., to study gain and loss patterns of specific classes of transcription factor binding sites [49], we do not recommend using events on the *D. pseudoobscura *branch.

## Conclusions

We have presented a novel method for generating benchmarks of non-coding sequence alignments, that relies on a spectrum of parameter values reflecting the genome-wide variation of those parameters. We have shown our benchmarks to accurately match the difficulty of aligning real data, by taking advantage of recent developments in measurement of alignment quality. Benchmark evaluations on *Drosophila *non-coding sequences suggest a greater accuracy of multiple alignment tools (in this domain) than previously reported, and points to a clear asymmetry in the handling of insertions versus deletions by most alignment tools.

## Methods

### *Drosophila *non-coding sequences and alignments

Whole-genome multiple alignments of *Drosophila *genome sequences (release 5) with 14 insects were downloaded from UCSC Genome Browser Database [1] and all exon positions were masked with symbol "N". An initial phylogeny was obtained from the AAA Drosophila website [50]. In cases where two sibling species are very close to each other, we chose one of them to include in this analysis leading to the following set of eight species: *D. melanogaster*, *D. simulans*, *D. yakuba*, *D. ananassae*, *D. pseudoobscura*, *D. willistoni*, *D. mojavensis*, and *D. grimshawi*. We extracted fragments of the genome-wide multiple alignments that have sequences for all eight species, whose minimum length is 1 Kbp, and which have less than 50% of their length masked (a total of 11867 alignment fragments with a total length of ~17 Mbp *D. melanogaster *sequences). The extracted alignments were used to estimate simulation parameter values, as described below. The distribution of HoT alignment quality scores [27] was computed from the sequences in these alignments by realigning them using Pecan [33].

### Non-coding sequence simulation by traditional method

Median branch lengths of a phylogenetic tree for eight *Drosophila *species were estimated from the multiple alignments described above, using Paml [51]. This phylogenetic tree is shown in Additional file 9. This tree was provided as input to the Dawg simulation program [18], with the evolutionary model being F81 [52], substitution to indel ratio set to 10:1 [21] and insertion to deletion ratio set to 1:1. We modified the Dawg program to model indel lengths as following a mixture of two geometric distributions, following [49], with parameters trained from the above multiple alignments and Indelign-based annotation of insertions and deletions. We also modified Dawg to allow it to simulate a sequence that includes so-called "conserved blocks", which are contiguous short segments of varying length, where the evolutionary rate is different from the rest of the sequence. Such conserved blocks were made to cover 20% of the sequence length on average, and their evolutionary rate was 10% of that outside the blocks [21]. The length distribution of the conserved blocks was obtained from Bergman and Kreitman [37]. The length of root sequences in the simulation was 10 Kbp [21] and the root sequence was sampled from a random pool of 10 Kbp non-coding segments of the *D. melanogaster *genome.

The estimated median branch lengths mentioned above reflect an average of the rates in conserved and non-conserved regions of real non-coding sequences, whereas the phylogeny input to Dawg by definition represents the substitution rate outside of blocks. Therefore, the branch lengths of the phylogeny were adjusted based on the specified coverage of conserved blocks and their evolutionary rates. Let *t*_*o *_be the overall evolutionary rate (the estimated branch length), *t*_*n *_be the unconstrained evolutionary rate (values provided to the simulation program), *α *be the fraction of sequence length that falls into conserved blocks, and *β *be the ratio of the evolutionary rate of conserved blocks to that outside blocks. Then we have:

### Distributions of simulation parameter values

The collection of branch lengths estimated from each fragment of multiple alignments described above, using Paml, was used to produce the distribution of branch lengths. As was done in the traditional simulation method, these branch lengths were adjusted by the above formula. The distributions of the ratio of substitutions to indels and the ratio of insertions to deletions were estimated from the above multiple alignments and Indelign-based annotation of insertions and deletions. The length distribution of indels was determined as in the traditional simulation method. To obtain the genome-wide distribution of the fraction of conserved blocks, we collected Phastcons [35] conservation scores from UCSC Genome Browser Database [1], scanned multiple alignments of *Drosophila *non-coding sequences and marked consecutive columns as a conserved block if the following two conditions hold: (i) they span at least 10 consecutive non-gapped columns and (ii) Phastcons scores of all columns are greater than or equal to 0.9 (see Additional file 10 for the distribution of the fraction of conserved blocks). The relative evolutionary rate of conserved blocks was set to the fixed value of 0.1, as in the traditional simulation. The length of a root sequence was set to 1 Kbp (average length of non-coding sequences in the extracted fragments of *Drosophila *alignments) and the root sequence was sampled from the *D. melanogaster *non-coding genome (see Additional file 11 for various descriptive statistics of traditional and new benchmarks).

### Evaluation of alignment programs on Pollard et al. benchmark

The benchmark generated by Pollard et al. [21] parameterizes each data set by a single value (substitutions per site) for the parameter, divergence distance. They provided estimate of this parameter value for the *D. melanogaster *and *D. pseudoobscura *pair (mean 2.4 and median 2.24) to link their simulations to the pair of species. They later updated this value in a new phylogeny http://www.danielpollard.com/trees.html. We used their divergence estimates from the latter phylogeny and the benchmark they prescribed for this level of divergence, and evaluated the alignment programs ourselves on this benchmark.

### Evaluation measures for indel annotation schemes

Indel Count Agreement is defined by the following formula, where *N*_*It *_and *N*_*Dt *_are true numbers of insertions and deletions, and *N*_*Ie *_and *N*_*De *_are predicted numbers of insertions and deletions.

Indel Ratio Agreement is defined by the following formula, with notation as above:

Indel Annotation Coverage is the fraction of indel positions on which the two annotations agree.

### Modification of Indelign

The time complexity of the Indelign program is exponential in the number of "conditionally dependent blocks" and this prohibits fast annotation of certain data sets with relatively large numbers of species [10]. To reduce the time complexity, when there are more conditionally dependent blocks than a predefined threshold, the alignment is heuristically partitioned by a block that has the smallest effect on the final indel annotation. This process is repeated until all dependent blocks with size greater than the threshold are resolved.

### Supplementary website

Source code for the modified Dawg and Indelign programs, phylogenetic trees, simulated sequences and their alignments, and computed alignments by six alignment tools are available from http://europa.cs.uiuc.edu/RealisticAlignmentBenchmarks/.

## Abbreviations

Indel: insertion and deletion; SPS: sum-of-pair score; CS: column score

## Authors' contributions

JK and SS conceived of the study, participated in its design, performed the analysis, and drafted the manuscript. JK developed the software and performed experiments. Both authors read and approved the final manuscript.

## Supplementary Material

Additional file 1 **Performance of multiple alignment tools compared by alignment sensitivity**. The scores were calculated by using all synthetic data sets (left panel), and by using only data sets where the expected number of insertions is two times more than the number of deletions or vice versa (middle and right panels respectively). Click here for file

Additional file 2 **Performance of multiple alignment tools compared by alignment specificity**. The scores were calculated by using all synthetic data sets (left panel), and by using only data sets where the expected number of insertions is two times more than the number of deletions or vice versa (middle and right panels respectively). Click here for file

Additional file 3 An example data set from the benchmark shown (in part) with true alignment (top panel) and alignments computed by each different programs. Click here for file

Additional file 4 Dependence of performance (sensitivity (left) and specificity (right)) of each alignment program on various descriptive statistics of the data sets. Click here for file

Additional file 5 **Performance of multiple alignment tools compared by alignment sensitivity of pairs of species**. The scores were calculated by using all synthetic data sets (left panel), and by using only data sets where the expected number of insertions is two times more than the number of deletions or vice versa (middle and right panels respectively). Click here for file

Additional file 6 **Performance of multiple alignment tools compared by alignment specificity of pairs of species**. The scores were calculated by using all synthetic data sets (left panel), and by using only data sets where the expected number of insertions is two times more than the number of deletions or vice versa (middle and right panels respectively). Click here for file

Additional file 7 Comparison of estimated alignment sensitivity and specificity, using Mlagan or Pecan, as obtained from the Pollard et al. [21] benchmark and from our benchmark. Click here for file

Additional file 8 Comparison of estimated alignment sensitivity and specificity as obtained from the Pollard et al. benchmark. Click here for file

Additional file 9 Phylogenetic trees and branch lengths in Newick format. Click here for file

Additional file 10 Genome-wide distribution of the fraction of conserved blocks estimated by using Phastcons conservation scores and multiple alignments of *Drosophila *non-coding sequences obtained from UCSC Genome Browser Database. Click here for file

Additional file 11 Descriptive statistics of traditional and new benchmarks. Click here for file
