# Supplementary material for: Towards realistic benchmarks for multiple alignments of non-coding sequences
Source: BMC Bioinformatics. 2010 Jan 26;11:54. doi: 10.1186/1471-2105-11-54 (PMC2823711; doi:10.1186/1471-2105-11-54)
Supplement: Additional file 1 — Performance of multiple alignment tools compared by alignment sensitivity. The scores were calculated by using all synthetic data sets (left panel), and by using only data sets where the expected number of insertions is two times more than the number of deletions or vice versa (middle and right panels respectively). [file 1471-2105-11-54-S1.DOC]

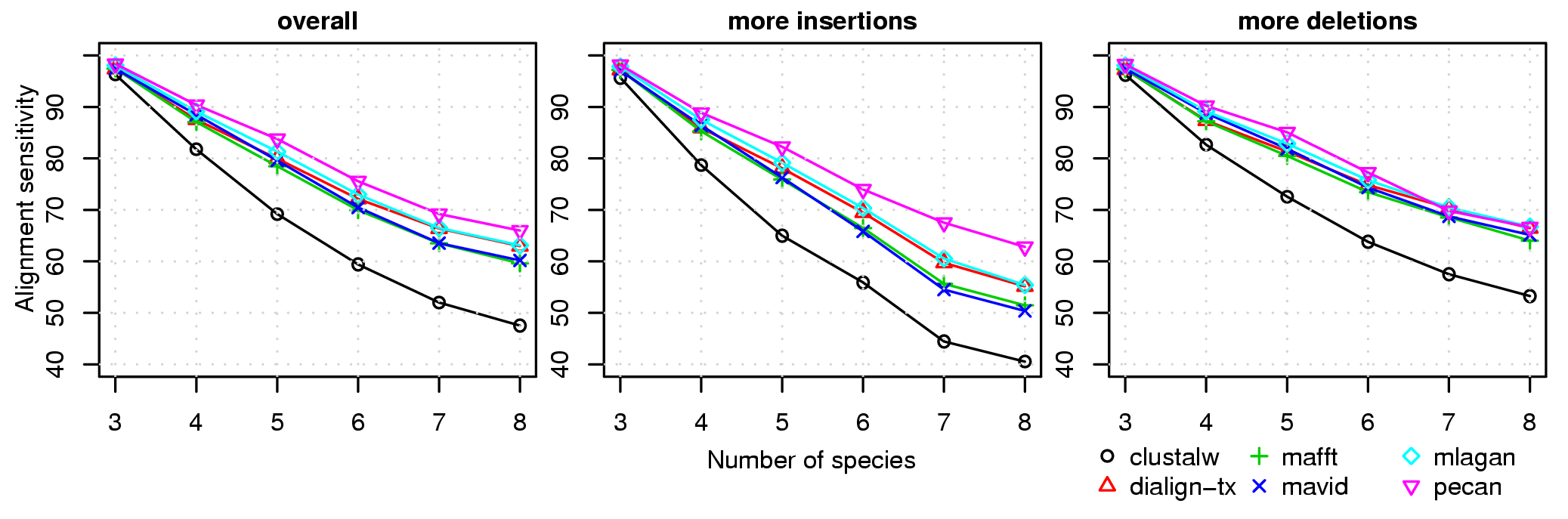


Figure S1. Performance of multiple alignment tools compared by alignment sensitivity. The scores were calculated by using all synthetic data sets (left panel), and by using only data sets where the expected number of insertions is two times more than the number of deletions or vice versa (middle and right panels respectively).
