# Supplementary material for: Towards realistic benchmarks for multiple alignments of non-coding sequences
Source: BMC Bioinformatics. 2010 Jan 26;11:54. doi: 10.1186/1471-2105-11-54 (PMC2823711; doi:10.1186/1471-2105-11-54)
Supplement: Additional file 3 — An example data set from the benchmark shown (in part) with true alignment (top panel) and alignments computed by each different programs. [file 1471-2105-11-54-S3.DOC]

True Alignment


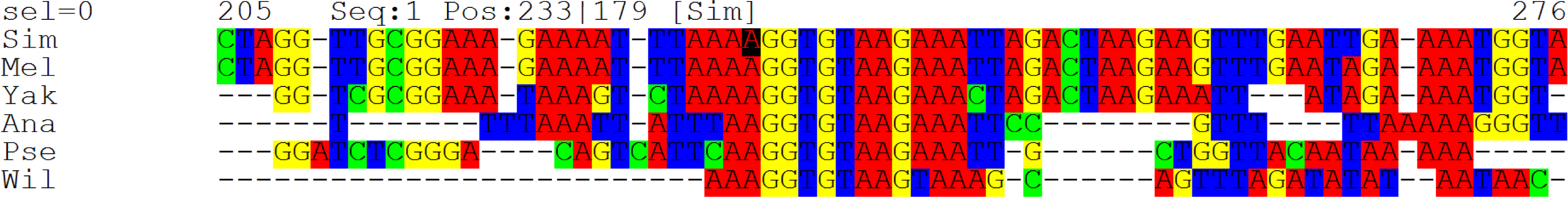


ClustalW Alignment


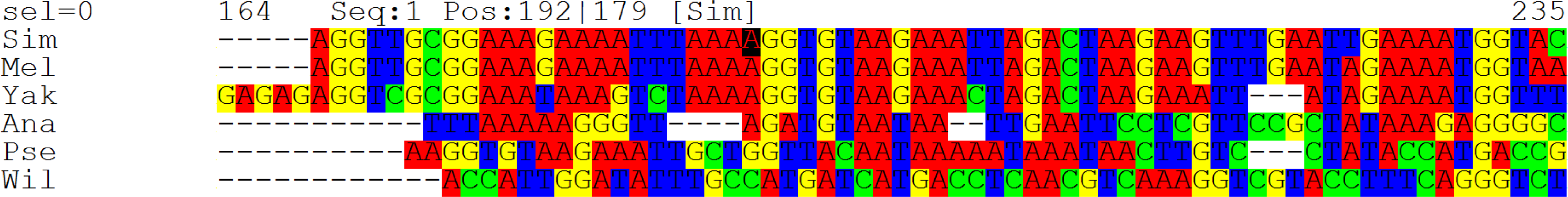


Dialign-TX Alignment


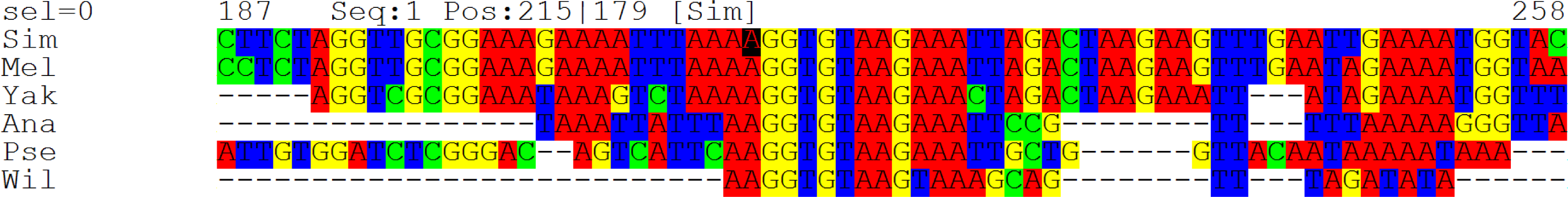


MAFFT Alignment


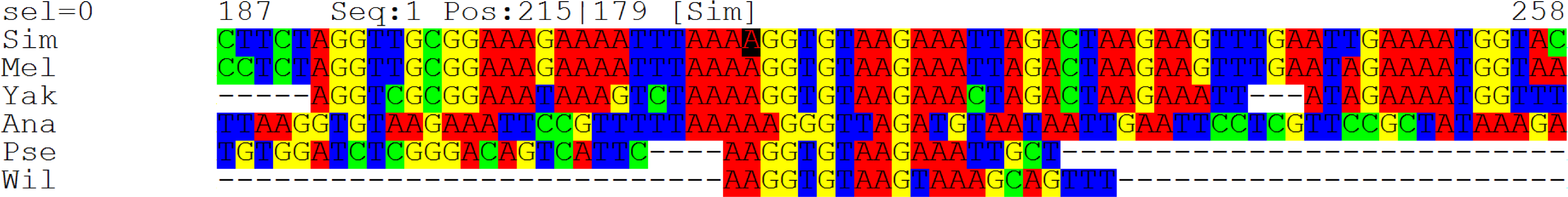


Mavid Alignment


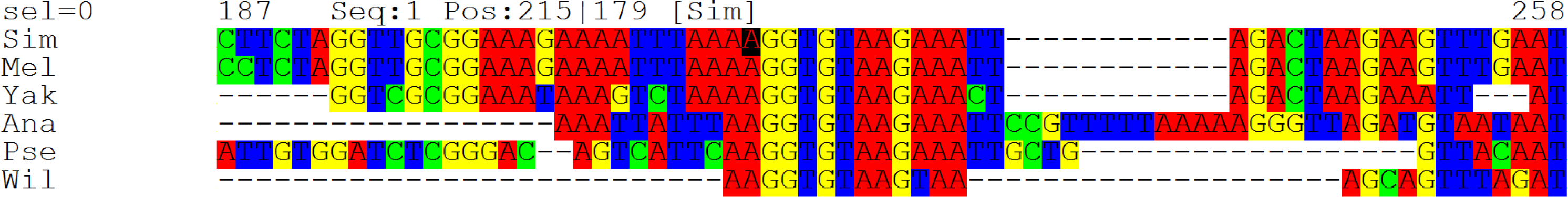


Mlagan Alignment


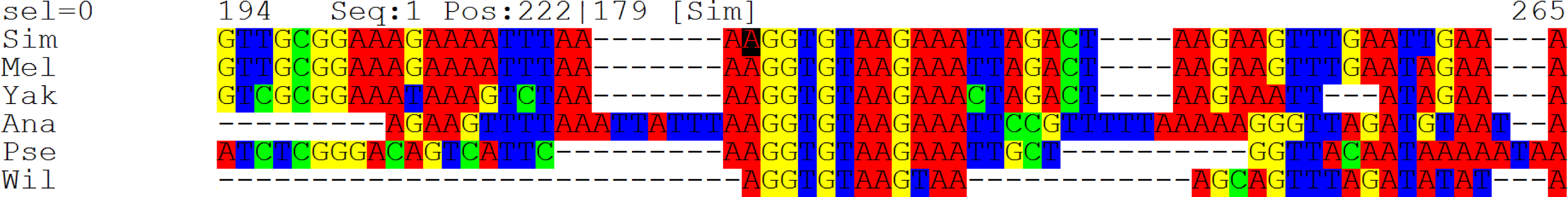


Pecan Alignment


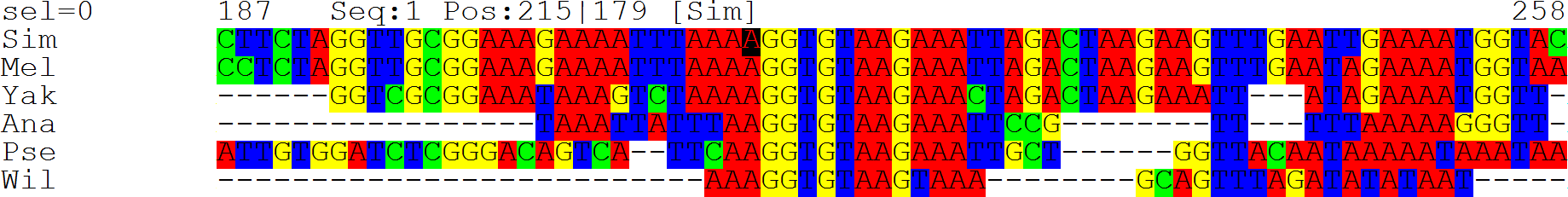


Figure S3. An example data set from the benchmark shown (in part) with true alignment (top panel) and alignments computed by each different program. The alignments were visualized using SeaView (http://pbil.univ-lyon1.fr/software/seaview.html).
