# Supplementary material for: Towards realistic benchmarks for multiple alignments of non-coding sequences
Source: BMC Bioinformatics. 2010 Jan 26;11:54. doi: 10.1186/1471-2105-11-54 (PMC2823711; doi:10.1186/1471-2105-11-54)
Supplement: Additional file 4 — Dependence of performance (sensitivity (left) and specificity (right)) of each alignment program on various descriptive statistics of the data sets. [file 1471-2105-11-54-S4.DOC]

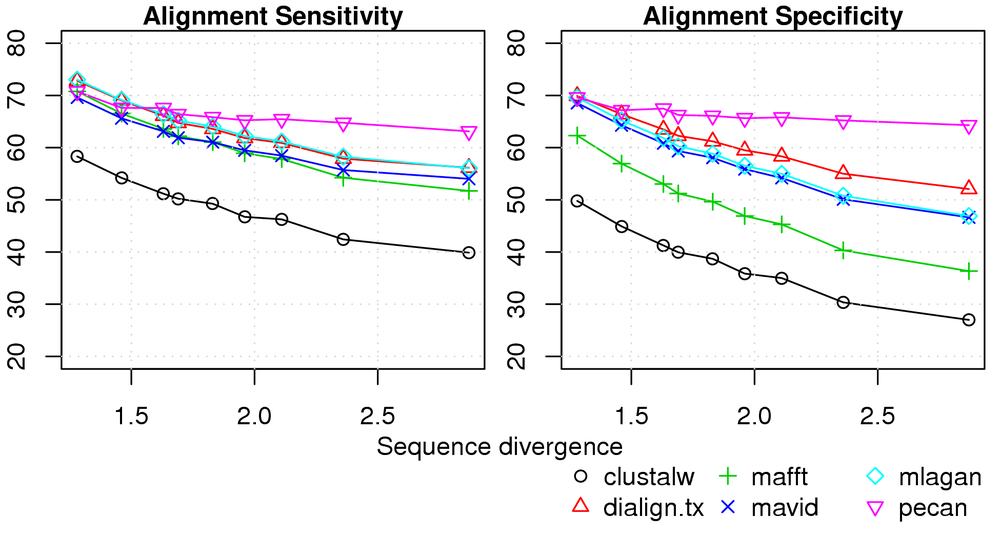


Figure S4-1. Dependence of performance (sensitivity (left) and specificity (right)) of each alignment program on sequence divergence in 8 species data sets. The sequence divergence was defined as the sum of branch lengths of the phylogeny used to generate each data set. (The phylogeny used to generate a data set is obtained from a single phylogeny *φ_0_* by scaling with a constant factor τ that is sampled from an empirical distribution.)


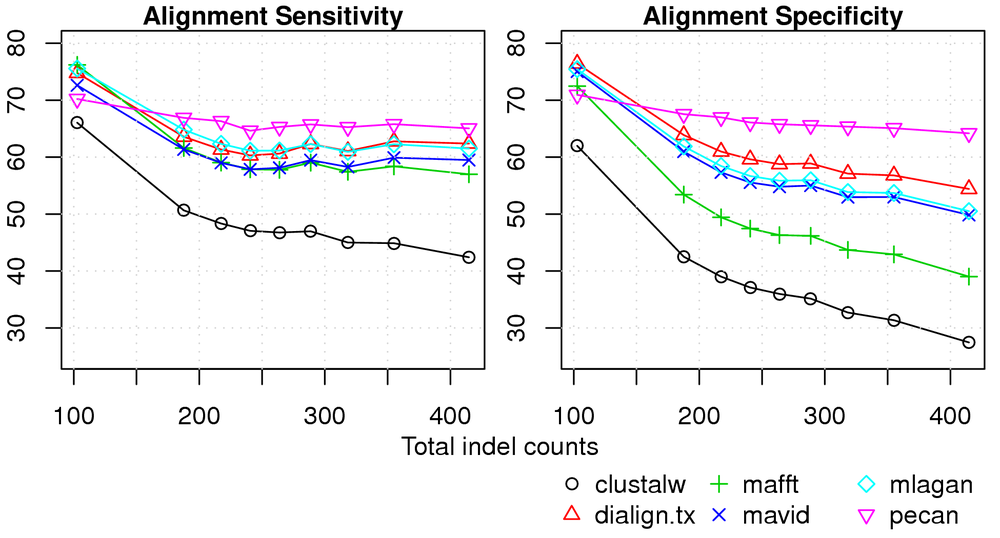


Figure S4-2. Dependence of performance (sensitivity (left) and specificity (right)) of each alignment program on total indel counts on terminal branches in 8 species data sets.


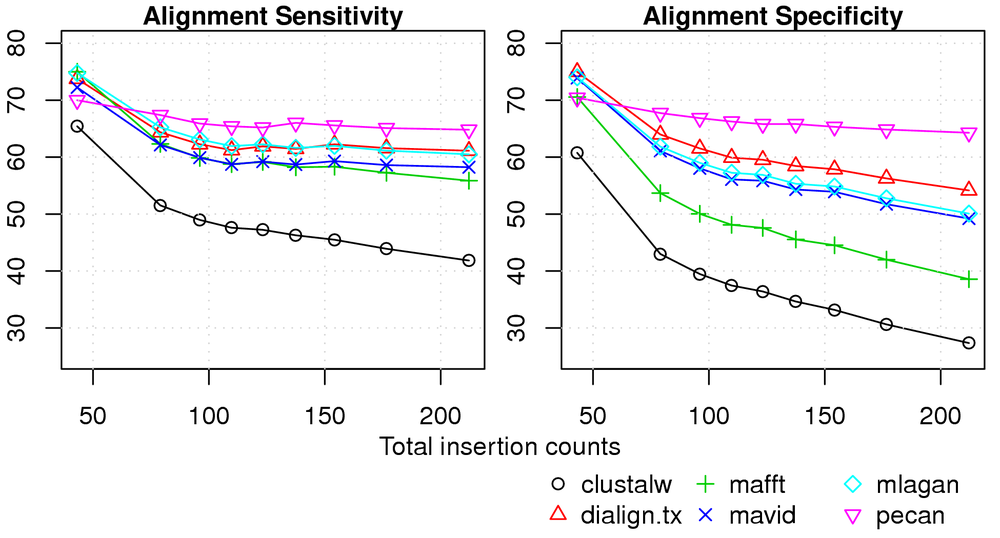


Figure S4-3. Dependence of performance (sensitivity (left) and specificity (right)) of each alignment program on total insertion counts on terminal branches in 8 species data sets.


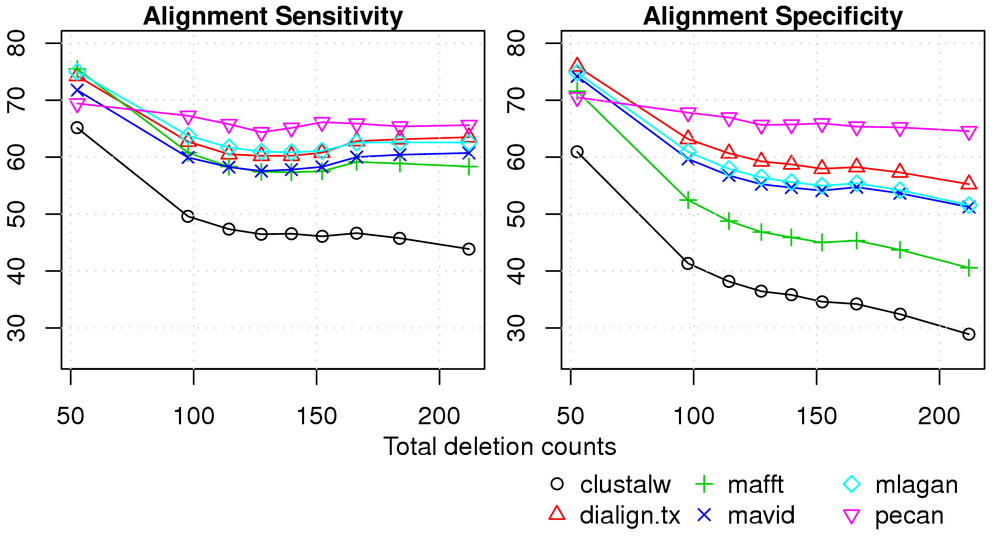


Figure S4-4. Dependence of performance (sensitivity (left) and specificity (right)) of each alignment program on total deletion counts on terminal branches in 8 species data sets.


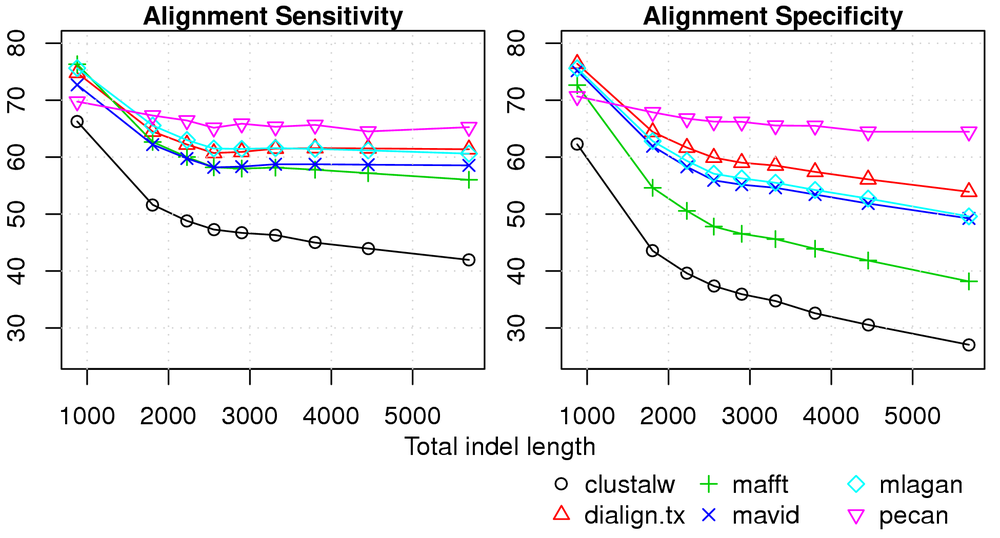


Figure S4-5. Dependence of performance (sensitivity (left) and specificity (right)) of each alignment program on total indel length on terminal branches in 8 species data sets.


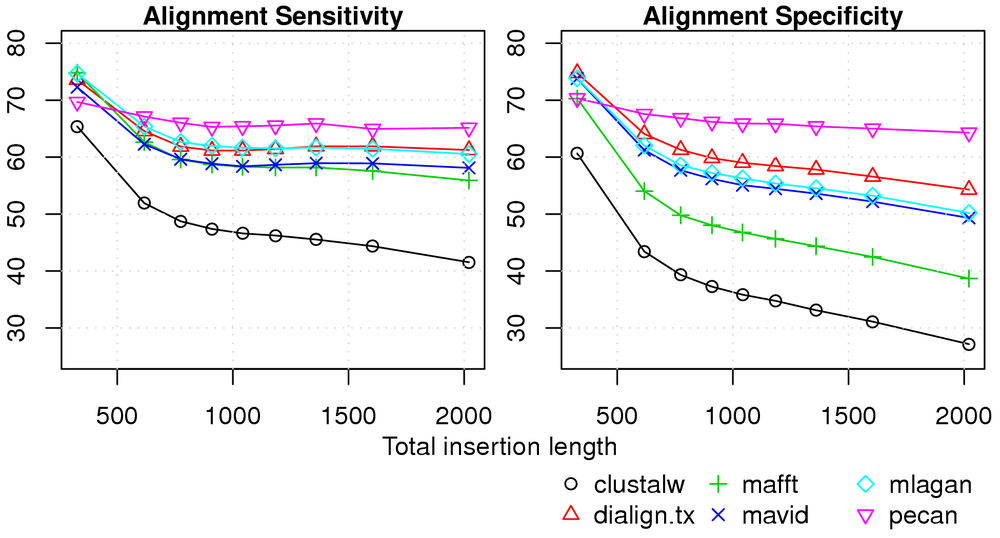


Figure S4-6. Dependence of performance (sensitivity (left) and specificity (right)) of each alignment program on total insertion length on terminal branches in 8 species data sets.


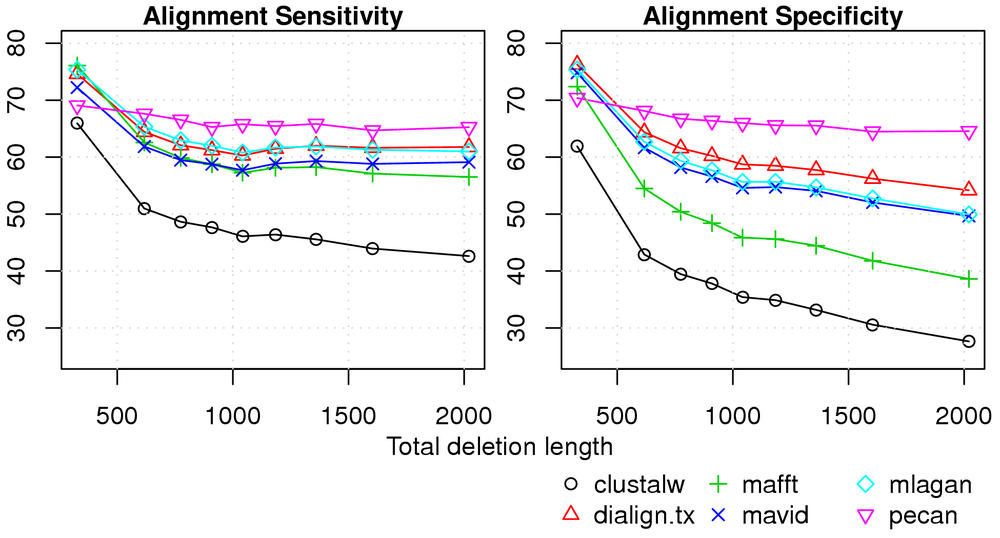


Figure S4-7. Dependence of performance (sensitivity (left) and specificity (right)) of each alignment program on total deletion length on terminal branches in 8 species data sets.
