# Supplementary material for: Towards realistic benchmarks for multiple alignments of non-coding sequences
Source: BMC Bioinformatics. 2010 Jan 26;11:54. doi: 10.1186/1471-2105-11-54 (PMC2823711; doi:10.1186/1471-2105-11-54)
Supplement: Additional file 7 — Comparison of estimated alignment sensitivity and specificity, using Mlagan or Pecan, as obtained from the Pollard et al. [21] benchmark and from our benchmark. [file 1471-2105-11-54-S7.DOC]

|  |  | **Pecan** | | | | **Mlagan** | | | |
| --- | --- | --- | --- | --- | --- | --- | --- | --- | --- |
| **Species** | **Div(P)^a^** | **Sen(P)** | **Sen(O)** | **Spe(P)** | **Spe(O)** | **Sen(P)** | **Sen(O)** | **Spe(P)** | **Spe(O)** |
| **D.sim** | 0.1243 | 99 | 99 | 98 | 99 | 98 | 99 | 98 | 99 |
| **D.yak** | 0.2850 | 97 | 98 | 96 | 97 | 96 | 97 | 95 | 97 |
| **D.ana** | 1.2763 | 36 | 80 | 55 | 76 | 39 | 78 | 32 | 72 |
| **D.pse** | 1.5337 | 33 | 73 | 53 | 69 | 35 | 68 | 27 | 63 |
| **D.gri** | 2.0002 | 33 | 50 | 53 | 48 | 34 | 46 | 24 | 39 |
| **D.moj** | 2.1586 | 33 | 50 | 52 | 47 | 34 | 46 | 23 | 39 |

Table S1. Comparison of estimated alignment sensitivity and specificity, using Mlagan or Pecan, as obtained from the Pollard et al. benchmark and from our benchmark. For each pair of species (*D. melanogaster* and the species named in column 1), our benchmark was constructed as described in text, while the Pollard et al. benchmark was obtained from [21], using the divergence estimate for that species pair as provided by the same authors from <http://www.danielpollard.com/trees.html>.

^a^Divergence time (synonymous substitutions per site)

Sen(P): sensitivity by using Pollard’s benchmarks

Sen(O): sensitivity by using our new benchmarks

Spe(P): specificity by using Pollard’s benchmarks

Spe(O): specificity by using our new benchmarks
