# Supplementary material for: Towards realistic benchmarks for multiple alignments of non-coding sequences
Source: BMC Bioinformatics. 2010 Jan 26;11:54. doi: 10.1186/1471-2105-11-54 (PMC2823711; doi:10.1186/1471-2105-11-54)
Supplement: Additional file 8 — Comparison of estimated alignment sensitivity and specificity as obtained from the Pollard et al. benchmark. [file 1471-2105-11-54-S8.DOC]

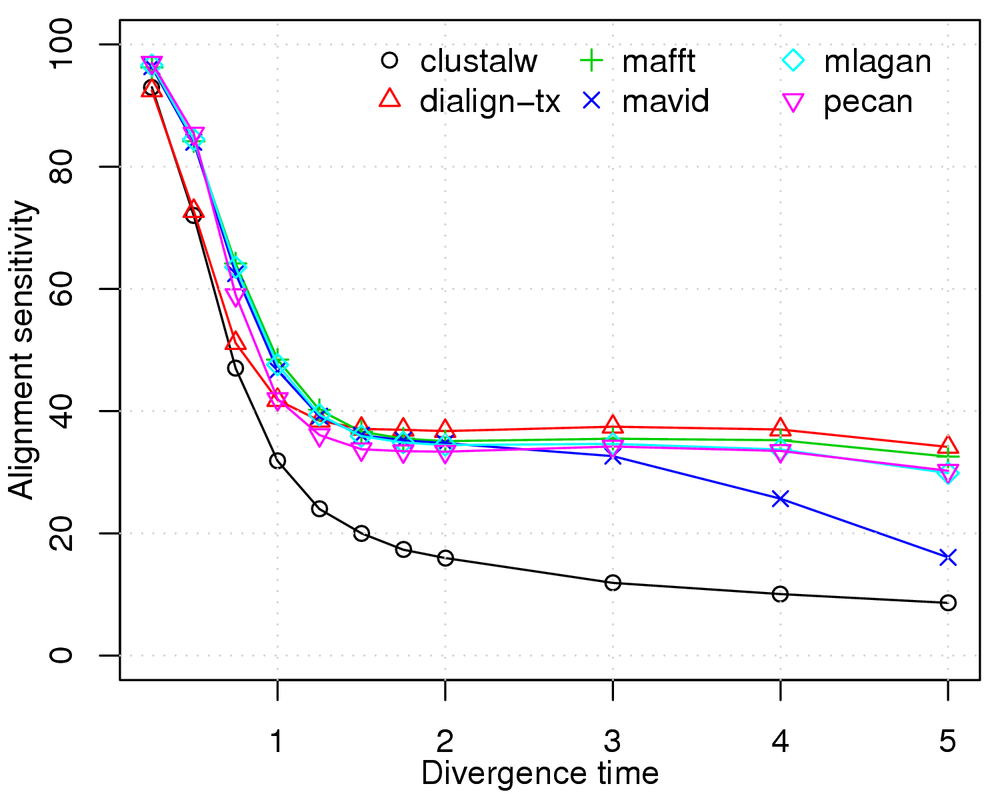

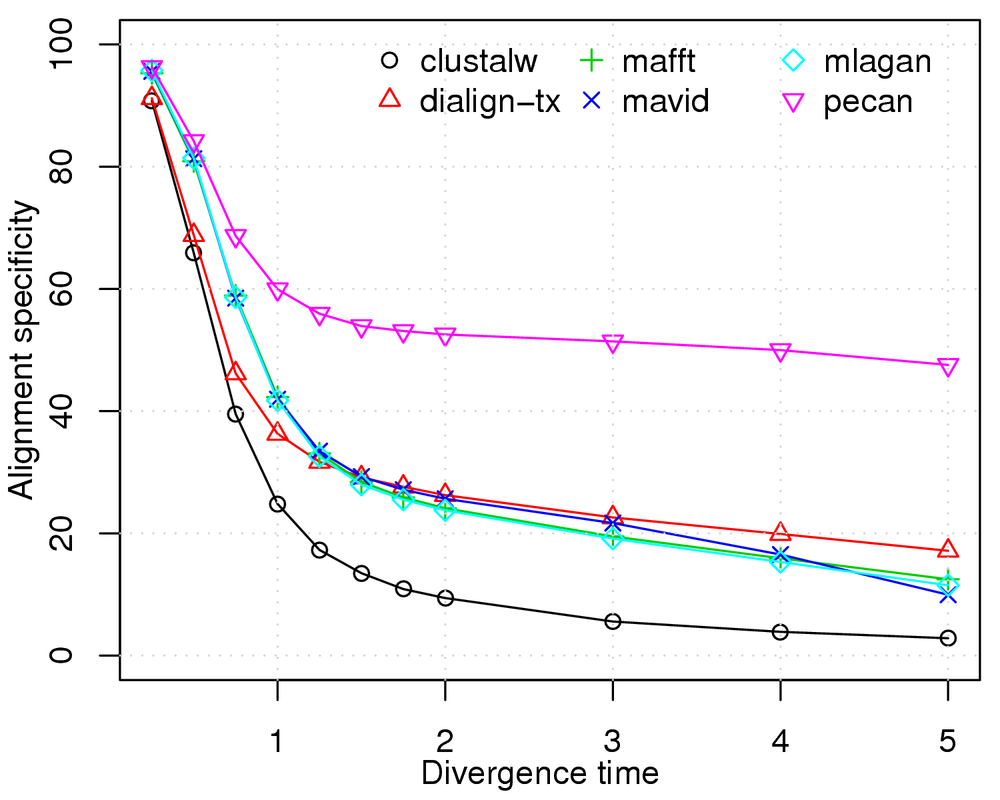


Figure S7. Comparison of estimated alignment sensitivity and specificity as obtained from the Pollard et al. benchmark. The Pollard et al. benchmark was obtained from [21].
