# Supplementary material for: Towards realistic benchmarks for multiple alignments of non-coding sequences
Source: BMC Bioinformatics. 2010 Jan 26;11:54. doi: 10.1186/1471-2105-11-54 (PMC2823711; doi:10.1186/1471-2105-11-54)
Supplement: Additional file 10 — Genome-wide distribution of the fraction of conserved blocks estimated by using Phastcons conservation scores and multiple alignments of Drosophila non-coding sequences obtained from UCSC Genome Browser Database. [file 1471-2105-11-54-S10.DOC]

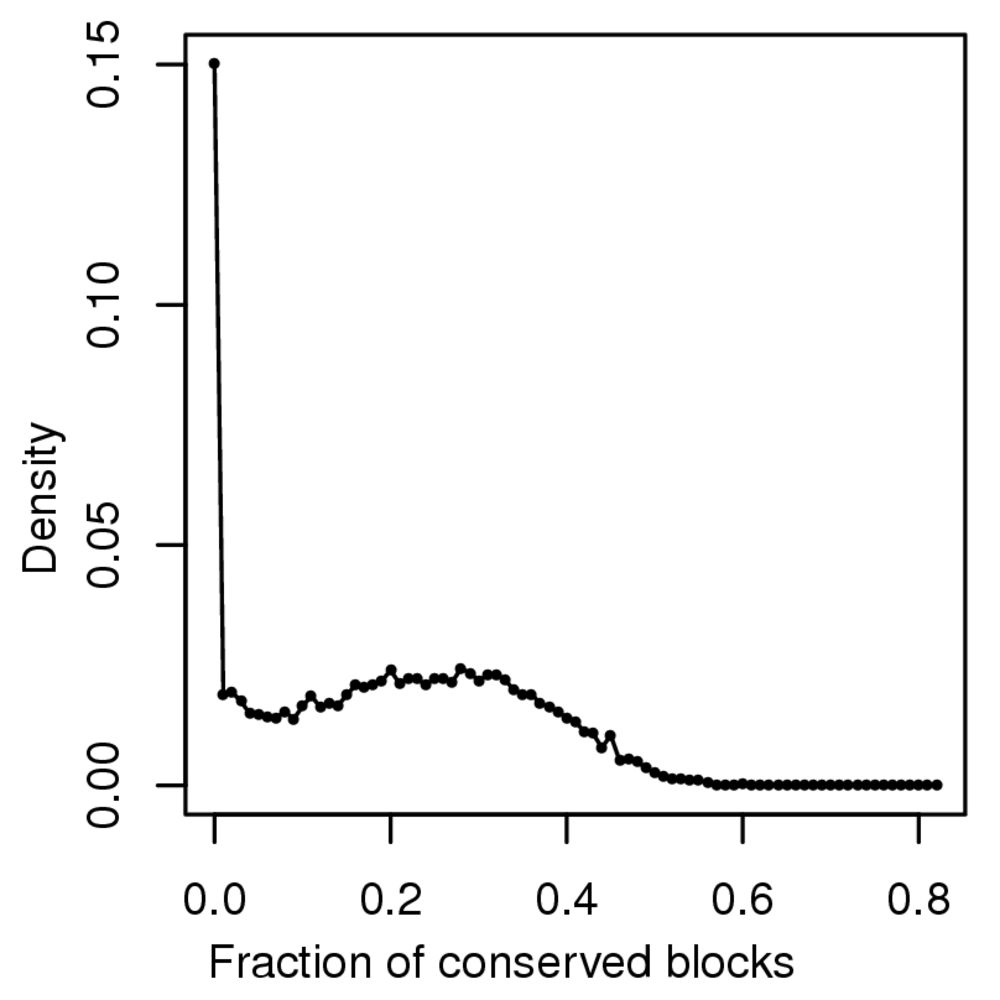


Figure S8. Genome-wide distribution of the fraction of conserved blocks estimated by using Phastcons conservation scores and multiple alignments of *Drosophila* non-coding sequences obtained from UCSC Genome Browser Database (see Methods for the criteria to identify conserved blocks).
