# Supplementary material for: Towards realistic benchmarks for multiple alignments of non-coding sequences
Source: BMC Bioinformatics. 2010 Jan 26;11:54. doi: 10.1186/1471-2105-11-54 (PMC2823711; doi:10.1186/1471-2105-11-54)
Supplement: Additional file 11 — Descriptive statistics of traditional and new benchmarks. [file 1471-2105-11-54-S11.DOC]

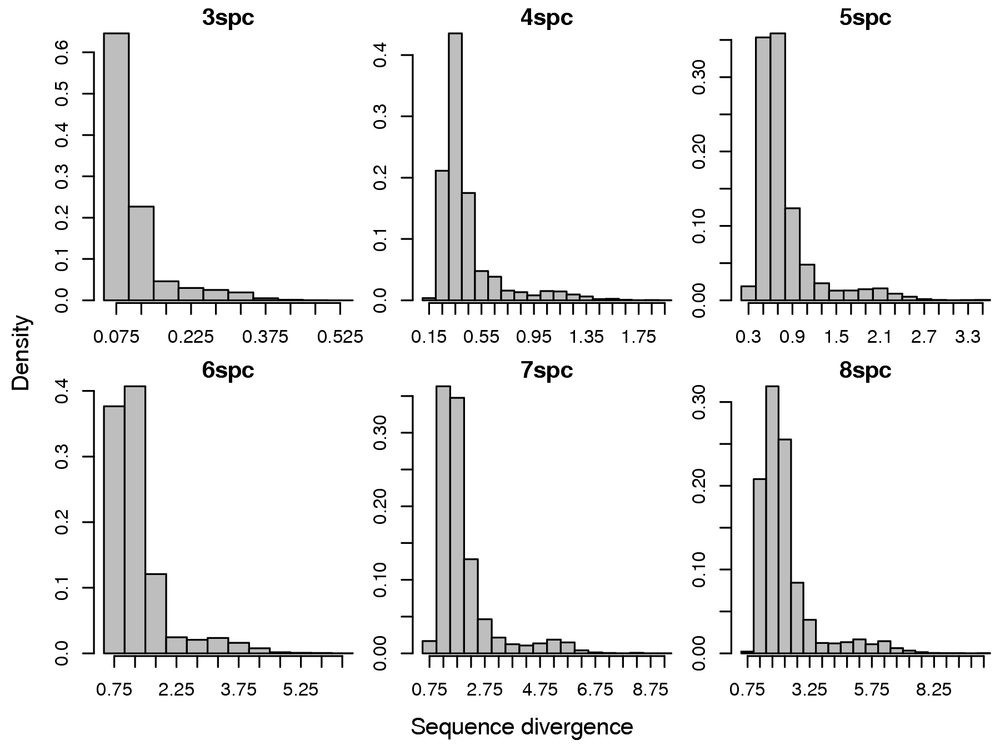


Figure S9-1. Sequence divergence of data sets in benchmark. For each data set (i.e., set of simulated “orthologous” sequences), we computed the sum of branch lengths of the phylogeny used to generate that data set. (The phylogeny used to generate a data set is obtained from a single phylogeny *φ_0_* by scaling with a constant factor τ that is sampled from an empirical distribution.) Shown is the histogram of this “sum of branch lengths” statistic, for benchmarks with 3,…,8 species.


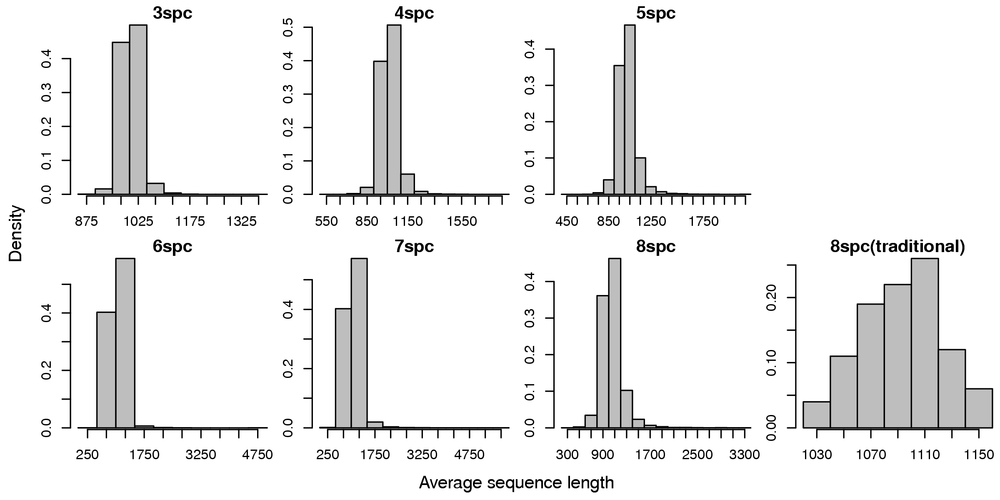


Figure S9-2. Average sequence length of data sets in benchmark. For each data set we computed the average length of sequences at the leaves of the phylogeny. (In each case, the root sequence was of length 1000 bp, but the leaf node sequences varied in length due to insertions and deletions accumulated during the simulated evolution.) Shown is the histogram of this statistic, for benchmarks with 3,…,8 species, and also a “traditional” benchmark with 8 species.


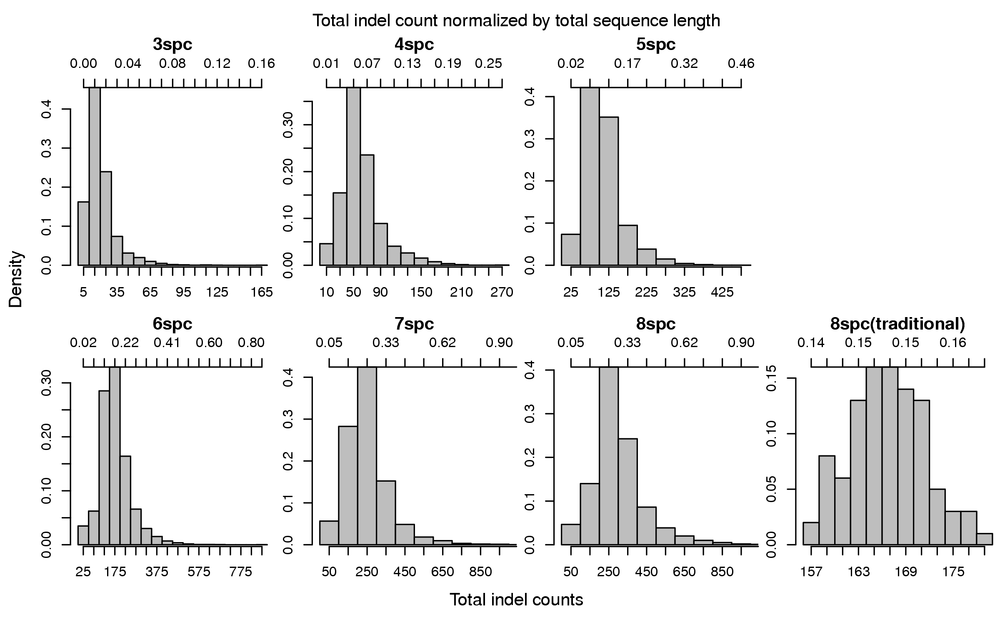


Figure S9-3. Total indel counts on terminal branches in data sets in benchmark. Shown is the histogram of this statistic, for benchmarks with 3,…,8 species, and also a “traditional” benchmark with 8 species. The x-axis shows both the total counts (bottom) and the total count normalized by total sequence length (top).


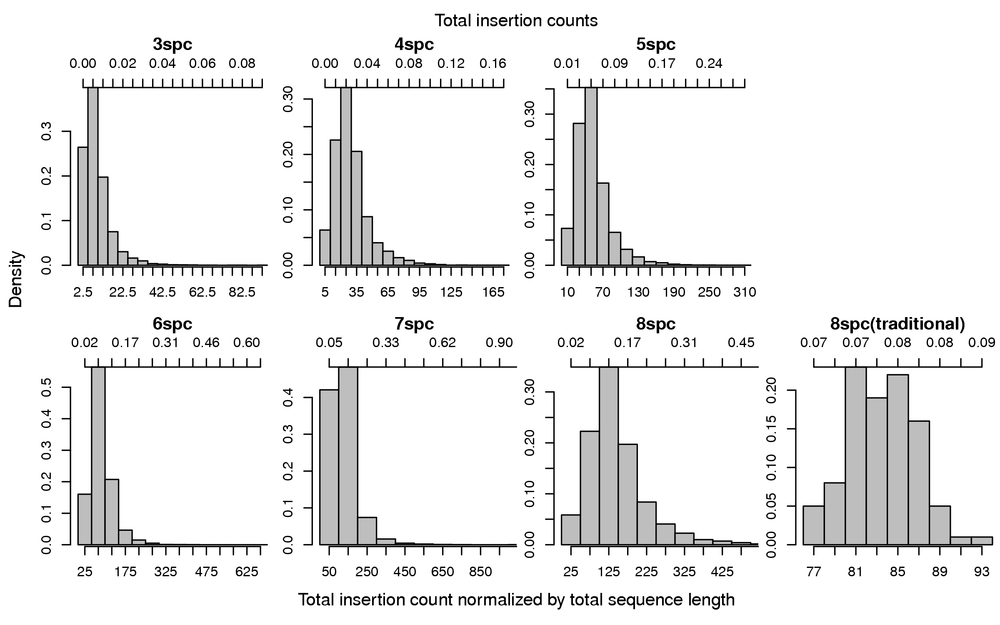


Figure S9-4. Total insertion counts on terminal branches in data sets in benchmark. Shown is the histogram of this statistic, for benchmarks with 3,…,8 species, and also a “traditional” benchmark with 8 species. The x-axis shows both the total counts (bottom) and the total count normalized by total sequence length (top).


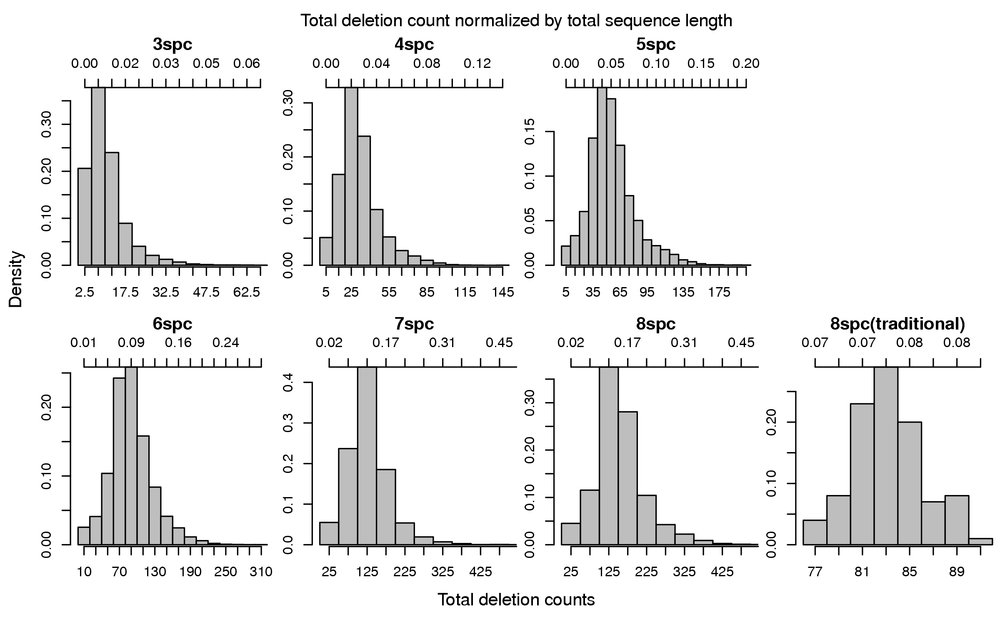


Figure S9-5. Total deletion counts on terminal branches in data sets in benchmark. Shown is the histogram of this statistic, for benchmarks with 3,…,8 species, and also a “traditional” benchmark with 8 species. The x-axis shows both the total counts (bottom) and the total count normalized by total sequence length (top).


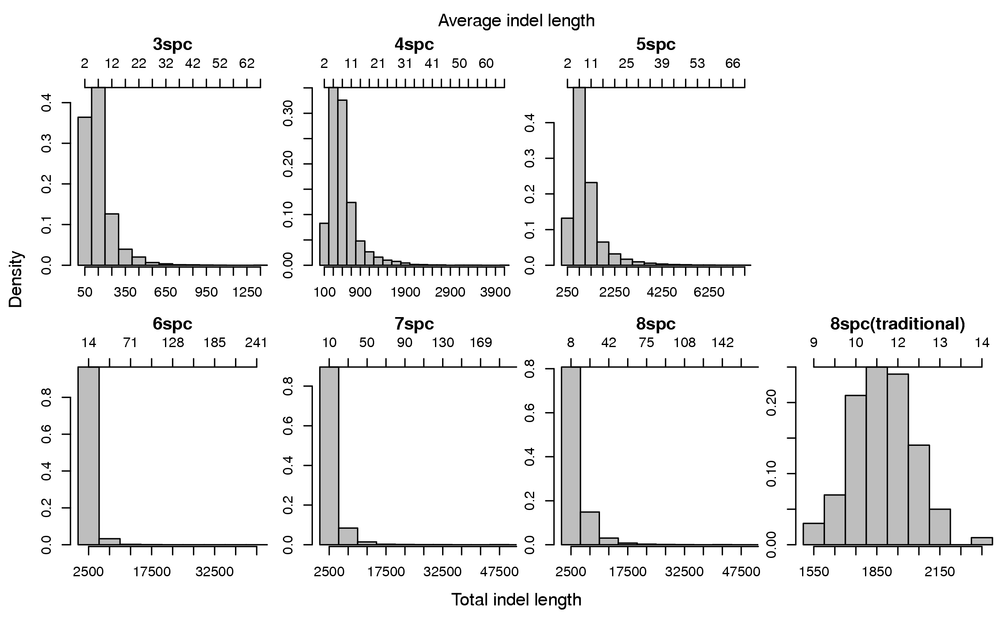


Figure S9-6. Total indel length on terminal branches in data sets in benchmark. Shown is the histogram of this statistic, for benchmarks with 3,…,8 species, and also a “traditional” benchmark with 8 species. The x-axis shows both the total length (bottom) and the average length (top).


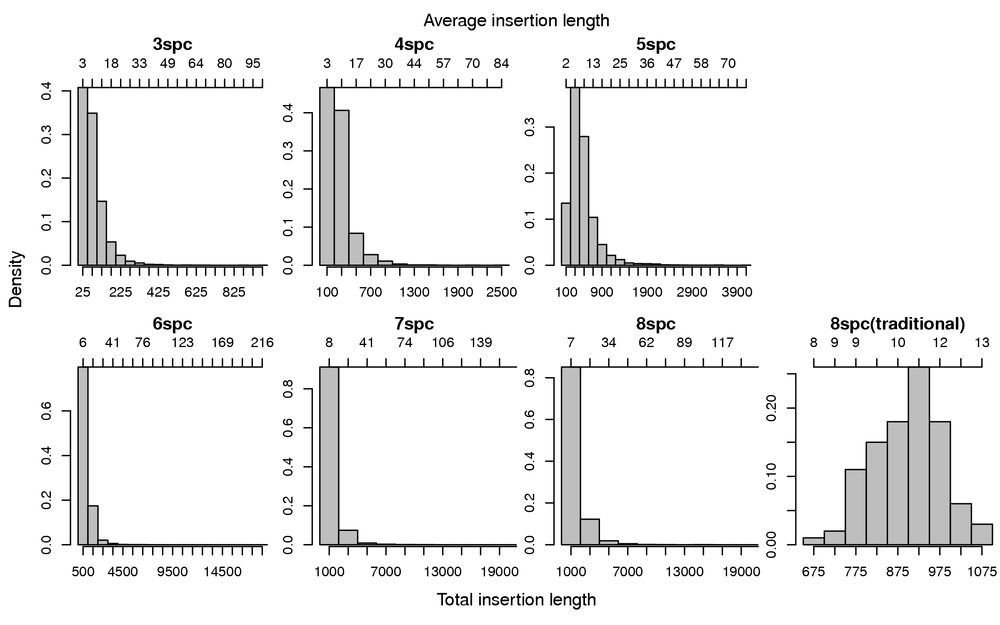


Figure S9-7. Total insertion length on terminal branches in data sets in benchmark. Shown is the histogram of this statistic, for benchmarks with 3,…,8 species, and also a “traditional” benchmark with 8 species. The x-axis shows both the total length (bottom) and the average length (top).


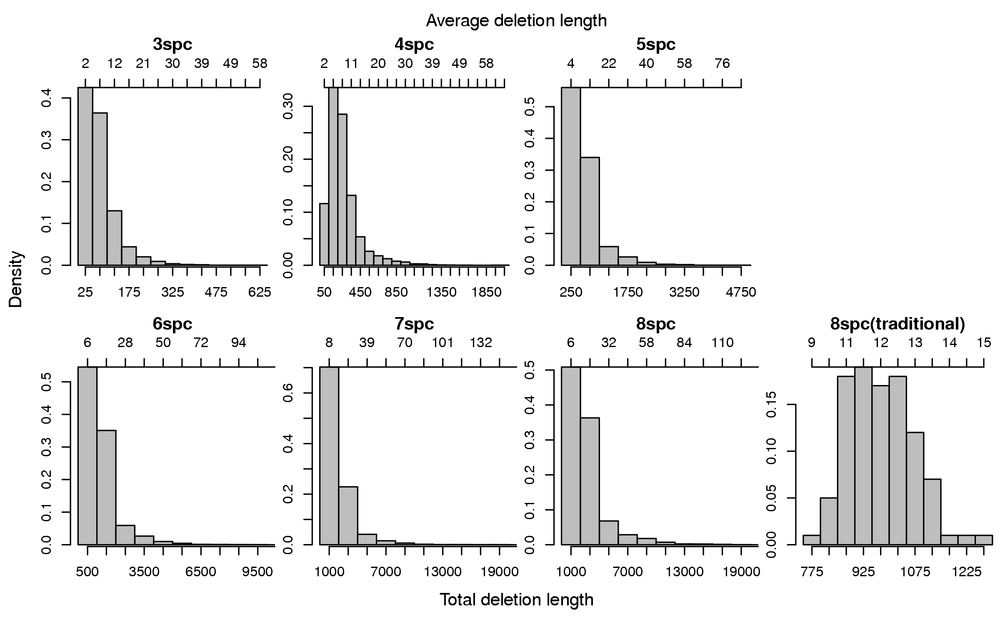


Figure S9-8. Total deletion length on terminal branches in data sets in benchmark. Shown is the histogram of this statistic, for benchmarks with 3,…,8 species, and also a “traditional” benchmark with 8 species. The x-axis shows both the total length (bottom) and the average length (top).
